# Characterization of Signal Sequences Determining the Nuclear/Nucleolar Import and Nuclear Export of the Caprine Arthritis-Encephalitis Virus Rev Protein

**DOI:** 10.3390/v12080900

**Published:** 2020-08-17

**Authors:** Marlène Labrecque, Claude Marchand, Denis Archambault

**Affiliations:** 1 Département des Sciences Biologiques, Université du Québec à Montréal, Montréal, QC H3C 3P8, Canada; labrecque.marlene@courrier.uqam.ca (M.L.); marchand.claude@uqam.ca (C.M.); 2 Centre d’Excellence en Recherche sur les Maladies Orphelines-Fondation Courtois (CERMO-FC), Université du Québec à Montréal, Montréal, QC H3C 3P8, Canada; 3 Département de Microbiologie, Infectiologie et Immunologie, Université de Montréal, Montréal, QC H3C 3J7, Canada; 4 Centre de Recherche en Infectiologie Porcine et Avicole (CRIPA), Université de Montréal, Montréal, QC H3C 3J7, Canada

**Keywords:** caprine arthritis-encephalitis virus, small ruminant lentivirus (SRLV), Rev protein, nuclear localization signal (NLS), nucleolar localization signal (NoLS), nuclear export signal (NES)

## Abstract

Caprine arthritis-encephalitis virus (CAEV), a lentivirus, relies on the action of the Rev protein for its replication. The CAEV Rev fulfills its function by allowing the nuclear exportation of partially spliced or unspliced viral mRNAs. In this study, we characterized the nuclear and nucleolar localization signals (NLS and NoLS, respectively) and the nuclear export signal (NES) of the CAEV Rev protein. These signals are key actors in the nucleocytoplasmic shuttling of a lentiviral Rev protein. Several deletion and alanine substitution mutants were generated from a plasmid encoding the CAEV Rev wild-type protein that was fused to the enhanced green fluorescent protein (EGFP). Following cell transfection, images were captured by confocal microscopy and the fluorescence was quantified in the different cell compartments. The results showed that the NLS region is localized between amino acids (aa) 59 to 75, has a monopartite-like structure and is exclusively composed of arginine residues. The NoLS was found to be partially associated with the NLS. Finally, the CAEV Rev protein’s NES mapped between aa 89 to 101, with an aa spacing between the hydrophobic residues that was found to be unconventional as compared to that of other retroviral Rev/Rev-like proteins.

## 1. Introduction

Caprine arthritis-encephalitis virus (CAEV) is a lentivirus of the *Retroviridae* family. CAEV mainly infects domestic goats worldwide but can also be found in sheep. The virus causes chronic inflammation and degenerative lesions in the articulation joints, brain, mammary gland and lung, resulting in clinical signs of arthritis, encephalitis, mastitis, pneumonia and significant weight loss [1]. The virus has monocytes/macrophages as target cells for infection and the major means of CAEV transmission is from mother to kids via colostrum and milk [1,2]. CAEV generates significant economic loss from the diminution in weight and in milk production in addition to premature culling [3]. Owing to the fact that there is currently no treatment or vaccine to circumvent CAEV-associated disease, control of the virus in herds is recommended and requires good breeding practices to restrict viral transmission [1,4].

The CAEV provirus genome (8.4–9.2 kb in length [4]) contains the *gag*, *pol* and *env* enzyme/structural proteins encoding genes common to all retroviruses in addition to the long terminal repeats (LTRs) at both ends of the genome. It also contains additional open reading frames (ORFs) that encode four auxiliary proteins, termed Vif, Vpr-like, Rtm and Rev [5,6,7]. The lentiviral Rev protein plays a key role in the regulation of viral expression by the nuclear export of partially spliced or unspliced viral transcripts to achieve the virus life replication cycle [8,9].

To ensure its function, the Rev protein must shuttle between the cytoplasm and the nucleus/nucleolus through mechanisms that are driven by nuclear/nucleolar localization signals (NLS/NoLS) and nuclear export signal (NES). Several pathways have been described for the nuclear import of proteins [10]. In the classical pathway, the cargo protein is recognized first by importin α and serves as a bridging factor to importin β. To achieve its action, importin α binds to a NLS present within the protein. The NLS is generally composed of basic amino acid (aa) residues (arginines and lysines) and can be monopartite or bipartite in structure. Monopartite-type NLSs are composed of a single cluster of aa and are categorized into five classes based on the aa composition and the interaction with importin α [11]. Bipartite-type NLSs are composed of two clusters of aa separated by a short or a long spacer region. As such, the bipartite NLSs are classified as short and long types, containing, respectively, a spacer region of 8 to 16 aa or 30 to 32 aa between the two aa motifs that compose the NLS [12].

After having reached the nucleus, the Rev protein targets partially spliced or unspliced RNAs by the recognition of a stem-loop structure present in these viral RNAs, the so-called Rev responsive element (RRE) [13]. Following the initial binding of a Rev monomer to high-affinity binding sites of the RRE, up to twelve Rev molecules bind and multimerize through cooperative protein–protein and protein–RNA interactions [8]. Then, the chromosome region maintenance 1 (CRM1) protein, also known as exportin 1, binds to a short leucine-rich sequence present within the Rev protein, the NES, in order to achieve the nuclear export of the Rev protein–viral RNA complexes to the cytoplasm. Rev then dissociates from the protein–RNA complex and the viral RNAs can exert their function [8,13,14,15,16].

The CAEV Rev protein is a 18-kDa (133-aa-long) phosphoprotein that is essential for replication of the virus [16,17,18]. The NLS and NES domains of the CAEV Rev protein were barely studied in a previous report [19]. Therefore, we wished to conduct an extensive analysis to fully characterize the NLS, NoLS and NES of the CAEV Rev protein. By using several mutant proteins, it was found that the CAEV Rev protein NLS has a monopartite-like structure, spanning aa 59 to 75 of the protein sequence (^59^RRR-RWLRGIR^75^), and that the NoLS is almost completely associated with the NLS, also located between aa 59 to 75 but with the addition of aa 62 and 63 (^59^RRRRR-RWLRGIR^75^). It was also found that the nuclear export of the CAEV Rev protein is CRM1-dependent. Finally, the NES of CAEV Rev was found to map between aa 89 to 101 of the protein sequence, with a Φ^0^xxxΦ^1^xxΦ^2^xxΦ^3^xΦ^4^ NES-type sequence.

## 2. Materials and Methods

### 2.1. Cell Cultures and Transfection

Bovine macrophages (BoMac) [20] and adenocarcinoma cervical cancer (HeLa) [21] cell lines, free of mycoplasma, as demonstrated using the e-Myco VALiD Mycoplasma PCR detection kit (iNtRON Biotechnology, Burlington, MA, USA), were maintained at 37 °C in a humidified atmosphere of 5% CO_2_ in Roswell Park Memorial Institute (RPMI) 1640 medium and Eagle’s minimum essential medium (EMEM), respectively. All cell culture media were supplemented with 10% fetal bovine serum (PAA Laboratories Inc., Etobicoke, ON, Canada). For microscopy analyses, BoMac and HeLa cells were plated on coverslips in 24-well plates to a cell density of ~50% confluence. For the Rev activity assay, HeLa cells were plated at the same cell density in 6-well plates. All cells were transfected the next day in duplicate with each of the plasmid constructs (0.5 μg in the 24-well plates and 1 μg in the 6-well plates) used in this study with the X-tremeGENE^TM^9 transfection reagent (Roche, Indianapolis, IN, USA), as recommended by the manufacturer.

### 2.2. Plasmid Constructs

The gene encoding the CAEV Rev wild-type (WT) protein of the Cork strain (GenBank accession number M63105.1) was synthesized and subcloned into the pEGFP-C1 expression vector by Biobasic Inc. (Markham, ON, Canada). This plasmid construct (pEGFP-CAEV Rev WT) was able to express the CAEV Rev WT protein in fusion with EGFP (EGFP-CAEV Rev WT). Six CAEV Rev mutant proteins (M1 to M6) containing internal deletions ranging from 18 aa to 35 aa were generated from the pEGFP-CAEV Rev WT construct using the Gibson assembly mastermix (New England Biolabs, Ipswich, MA, USA). Alanine substitution mutants were introduced into the EGFP-CAEV Rev WT protein by PCR site-directed mutagenesis, using appropriate primers. To generate the pEGFP-NLS CAEV construct, the nucleic acids encoding aa 57 to 82 of the CAEV Rev WT protein, associated with a putative NLS, were amplified by PCR and cloned into the pEGFP-C1 plasmid. Briefly, the NLS was amplified by PCR with appropriated primers containing XhoI and EcoRI restriction sites. The fragments were purified, digested with XhoI and EcoRI and then ligated into the pEGFP-C1 digested with the same restriction enzymes. The EGFP-βGal and EGFP-βGal-NLS constructs were similarly generated using the βGal-encoding sequence derived from pSV-βGal (Clontech, Mountain View, CA, USA). All constructs in this study were validated by DNA sequencing through the McGill University Sequencing Services (Montréal, QC, Canada).

### 2.3. Microscopy Analysis

Following transfection with pEGFP-CAEV Rev WT or each mutant, cells were incubated for 24 h. Where indicated, cells were incubated in the presence of 5 nM of leptomycin B (LMB) 5 h prior to cell fixation. Cells were washed with phosphate-buffered saline solution (PBS), pH 7.3, and then were incubated for 15 min at room temperature with 4% paraformaldehyde in PBS. For the immunostaining, cells were permeabilized with 0.2% Triton X-100 for 10 min at room temperature and blocked for 1 h at 37 °C with 4% bovine serum albumin in PBS. Afterwards, the cells were incubated for 1 h at 37 °C with rabbit polyclonal IgG primary anti-C23 (nucleolin), washed with PBS and then incubated with Alexa 647-labeled anti-rabbit secondary antibodies (Santa Cruz Biotechnology, Inc., Dallas, TX, USA) for 1 h at room temperature. Finally, the nuclei were stained with 4,6-diamidino-2-phenylindole (DAPI; Invitrogen, Carlsbad, CA, USA) and coverslips were mounted on glass slides using the ProLong Gold antifade reagent (Invitrogen, Carlsbad, CA, USA). Cells were observed by confocal laser scanning microscopy (CLSM) using a Nikon A1 confocal system plus a 60× oil immersion objective. The images were then analyzed with the NIH ImageJ 1.62 public domain software. The mean fluorescence was determined in the cytoplasm (Fc), the nucleus (Fn) and the nucleoli (Fno). The results were expressed as nuclear/cytoplasmic (Fn/c) and nucleolar/nuclear (Fno/n) fluorescence ratios by using the equations Fn/c = (Fn − Fb)/(Fc − Fb) or Fno/n = (Fno − Fb)/(Fn − Fb), where Fb refers to the background fluorescence [22]. All data shown represent the general expression pattern observed in 30 cells analyzed from three independent experiments (10 analyzed cells per experiment).

### 2.4. CAT Assay

The CAEV Rev nuclear activity was quantified in transient transfection cell assays using a pDM138-based CAEV Rev chloramphenicol acetyltransferase (CAT) reporter construct containing the CAEV RRE (pRRE-CAEV) and developed according to our protocol [6]. The assay was conducted in HeLa cells since the transfection efficiency was high in these cells and resulted in an important expression of the CAEV Rev protein. The cDNA of the minimal region of CAEV RRE [18] was synthetized by BioBasics (Markham, ON, Canada). The cDNA was then amplified to introduce ClaI restriction sites at both ends and was subsequently cloned into the ClaI site of plasmid pDM138 [23] to generate pRRE-CAEV. HeLa cells were seeded in 6-well plates and cotransfected with 0.5 μg of empty pEGFP-C1, or each of the pEGFP constructs encoding either the CAEV Rev WT protein or each of the CAEV Rev mutant proteins, and 0.5 μg of pRRE-CAEV. At 48 h after transfection, the cells were harvested and lysed with lysis buffer (furnished in the CAT enzyme-linked immunosorbent assay kit (CAT-ELISA) kit; Roche, Penzberg, Germany). In each test, 50 μg of total cellular protein was assessed using the CAT-ELISA kit. The data were normalized to the level of EGFP-CAEV Rev protein expression detected via immunoblotting, as described elsewhere [24]. All data shown were obtained from three independent experiments, with triplicate samples per experiment. The results were expressed as the mean ratio of EGFP-Rev WT and each mutant CAT expression to the basal expression of pRRE-CAEV in the presence of EGFP alone. To determine the activity in the Rev(1.4)-EGFP nuclear export assay (see below), the pDM128 plasmid construct containing the HIV-1 RRE (pRRE-HIV) was used [25]. The same experimental steps used in the CAEV Rev nuclear activity were applied, the cells being cotransfected with 0.5 μg of either empty pEGFP-C1, Rev(1.4)-NES3-EGFP, Rev(1.4)-CAEVNESWT-EGFP or each of the Rev(1.4)-CAEVNES-EGFP mutant constructs (see below) and 0.5 μg of pRRE-HIV.

### 2.5. Immunoblotting

To assay protein expression levels via the ImageJ software for CAT-ELISA data normalization, 30 μg from each total cell lysate was separated by 12% sodium dodecyl sulfate-polyacrylamide gel electrophoresis (SDS PAGE). Following transfer, nitrocellulose membranes were blocked with 5% non-fat dry milk in PBS Tween 0.05% (PBS-T) for 1 h at room temperature. Mouse monoclonal primary antibodies specific to EGFP (Santa Cruz Biotechnology, Inc., B-2 clone, #sc-9996) or glyceraldehyde 3-phosphate dehydrogenase (GAPDH; Santa Cruz Biotechnology, Inc., G-9 clone, #sc-365062) were added for an additional incubation period of 1 h at room temperature. The membranes were washed in PBS-T and were incubated 1 h at room temperature with, as the secondary antibody, anti-mouse horseradish peroxidase-conjugated IgGs (#31430, Thermo Fisher, Waltham, MA, USA). All antibodies used were diluted in PBS-T containing 5% non-fat dry milk. Finally, the signal was detected by enhanced chemiluminescence with a Fusion FX7 apparatus (Vilber, Collégien, France).

### 2.6. Rev(1.4)-EGFP Nuclear Export Assay

To determine the aa essential for the CAEV Rev protein export, the Rev(1.4)-EGFP nuclear export assay was used [26]. The plasmid construct used in this assay, pRev(1.4)-EGFP, contains the whole HIV-1 Rev protein without the NES. Therefore, a predicted NES sequence can be cloned into the plasmid to promote the nuclear export of the HIV-1-EGFP fusion protein. The NES-deficient Rev(1.4)-EGFP and Rev(1.4)-NES3-EGFP (a construct that contains the intact HIV-1 Rev NES sequence) plasmids were kindly provided by Dr Beric R. Henderson (University of Sydney, Sydney, Australia) [9]. Alanine substitution NES mutant sequences were derived from the predicted CAEV Rev NES sequence by using complementary synthetic oligonucleotides that were ligated into the compatible ends of BamHI- and AgeI-digested Rev(1.4)-EGFP plasmid. After validating all mutant constructs by sequencing, HeLa cells were transfected with Rev(1.4)-EGFP (negative control), Rev(1.4) NES3-EGFP (positive control) or plasmids containing the NES sequence of CAEV Rev WT or each of the CAEV Rev NES mutated sequences. The cells were incubated for 24 h and were left untreated or exposed to both cycloheximide (CHX; 10 μg/mL) and actinomycin D (ActD; 5 μg/mL) 3 h prior to fixation. Finally, cells were fixed, counterstained with DAPI and the coverslips were mounted on glass slides using ProLong Gold antifade reagent (Thermo Fisher). Cells were imaged by CLSM and analyzed as described above. All data shown represent the general expression pattern observed in 30 cells analyzed from three independent experiments (10 analyzed cells per experiment).

### 2.7. Statistics

The results shown in this study were expressed as the mean values plus the standard deviation (SD). The software GraphPad Prism 7 (San Diego, CA, USA) was used to generate the statistical analyses. To compare data from two group means, Student’s T-test was used and, where specified, data were corrected with the Holm–Sidak method for multiple comparison of the means. To compare the mean of each CAEV Rev mutant to the mean of the CAEV Rev WT protein, a one-way ANOVA followed by a post-hoc Dunnett’s test (ANOVA Dunnett’s test) were used. Finally, to compare the means of different groups, a one-way ANOVA followed by a post-hoc Tukey’s multiple comparison test (ANOVA Tukey’s multiple comparison test) were used.

## 3. Results

### 3.1. The CAEV Rev Protein Localizes to the Cytoplasm, Nucleus and Nucleoli of Transfected Cells

**Figure 1:**
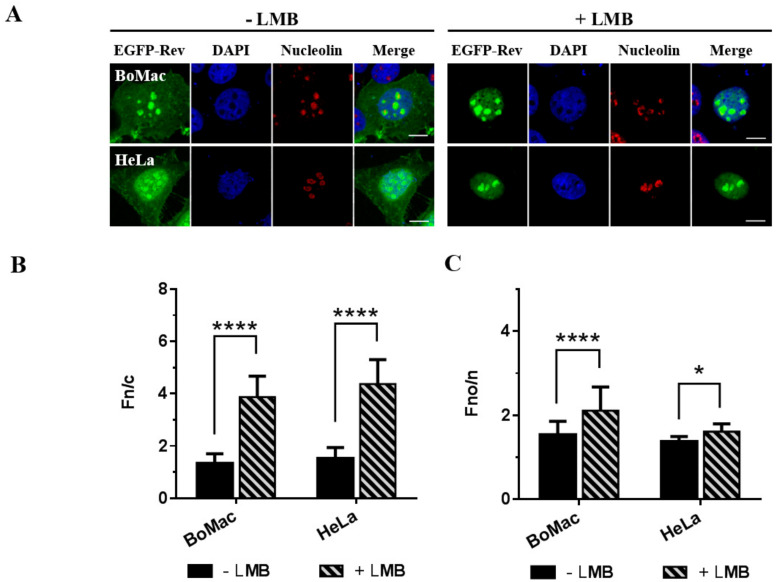
The EGFP-CAEV Rev WT fusion protein localizes to the cytoplasm, nucleus and nucleoli of transfected BoMac and HeLa cells. (**A**) Microscopic analyses of EGFP-CAEV Rev WT fusion protein in BoMac and HeLa cells. Cells were transfected with pEGFP-CAEV Rev WT for 24 h and were treated, where mentioned, with 5 nM of leptomycin B (LMB) 5 h prior to cell fixation and permeabilization. Cells were then immunostained for nucleolin detection, as seen in red, and the nucleus was counterstained with DAPI, as seen in blue. Expression of EGFP-Rev, as seen in green, was visualized by CLSM at 60× magnification. The images are representative of the expression pattern observed in cells from three independent experiments. The merge images represent the superposition of EGFP-Rev, DAPI and nucleolin, and the white bars represent a length of 10 μM. The calculated Fn/c (**B**) and Fno/n (**C**) ratios were expressed as means ± SD from three independent experiments, in which 10 cells were analyzed for each of them (*n* = 30). Using Student’s T-test corrected with the Holm–Sidak method for multiple comparison of the means, each cell line was analyzed for significant differences, which are indicated by * (*p* < 0.05) and **** (*p* < 0.0001). Using ANOVA and Tukey’s multiple comparison test, the Fn/c and Fno/n ratios measured for the BoMac cells were compared with those of the HeLa cells, regardless of the LMB treatment. No significant differences were observed between these cells.

To assess the subcellular distribution of the CAEV Rev protein by microscopy, BoMac and HeLa cells were transfected with the pEGFP-CAEV Rev WT vector. This plasmid encodes the CAEV Rev protein fused to enhanced green fluorescent protein (EGFP). As shown in Figure 1A (−LMB panel), the EGFP-CAEV Rev WT protein showed diffuse localization in the cytoplasm and the nucleus of both BoMac and HeLa cells. Moreover, the CAEV Rev protein readily accumulated in the nucleoli, as shown by the colocalization of the protein with the nucleolar marker nucleolin. These results were supported by the calculated Fn/c and Fno/n ratios (Figure 1B,C, −LMB bars). No significant differences were observed between the BoMac and HeLa cells’ Fn/c and Fno/n ratios. A previous study showed that the CAEV Rev protein localizes in the cytoplasm and the nucleus, with a strong concentration in the nucleoli of CAEV-infected goat synovial membrane cells [16]. Therefore, results of the EGFP-CAEV Rev protein subcellular localization obtained in the BoMac and Hela cells are similar to what was previously described in cells infected with CAEV.

### 3.2. Leptomycin B Blocks the Nuclear Export of the CAEV Rev Protein

All retroviral Rev and Rev-like proteins characterized so far rely on their interaction with CRM1 for their export from the nucleus to the cytoplasm [9,25,27,28,29,30]. To determine whether the nuclear export of the CAEV Rev protein is CRM1-dependent, the cells were transfected with pEGFP-CAEV Rev WT and treated with LMB 5 h prior to cell fixation for microscopy analysis. LMB is a potent inhibitor of CRM1 as it blocks the interaction between the latter and the NES present in a protein [31]. In presence of LMB (Figure 1A, +LMB panel), the CAEV Rev WT protein was exclusively localized in the nucleus and nucleoli of BoMac and HeLa cells. This increase in the nuclear/nucleolar accumulation was confirmed by the Fn/c and Fno/n calculated ratios (Figure 1B,C, +LMB bars). Thus, we concluded that CAEV Rev protein nuclear export is CRM1-dependent.

### 3.3. The Subcellular Localization Varies between CAEV Rev Deletion Mutant Proteins

**Figure 2:**
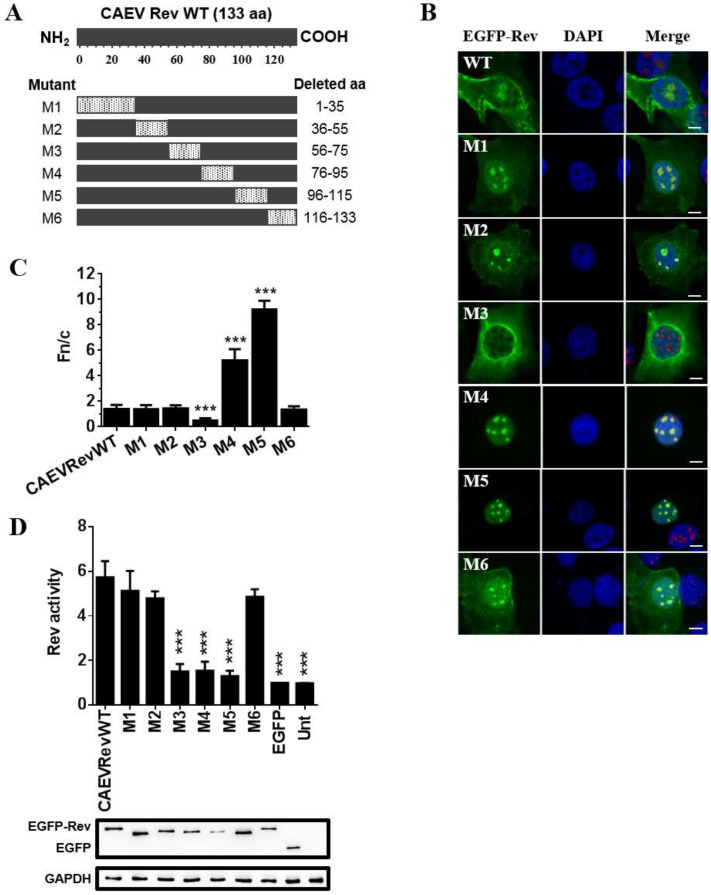
The amino acids between positions 56 to 115 are involved in the nuclear localization and nuclear export of the CAEV Rev protein. (**A**) CAEV Rev deletion mutants (M1 to M6) were generated from pEGFP-CAEV Rev WT using Gibson assembly mastermix. (**B**) Microscopic analyses of the CAEV Rev mutants M1 to M6 compared to the CAEV Rev WT. BoMac cells were transfected with pEGFP-CAEV Rev WT or each of the pEGFP-CAEV Rev mutants for 24 h and fixed. The nucleus was counterstained with DAPI, as seen in blue. Expression of EGFP-Rev, as seen in green, was visualized by CLSM at 60× magnification. The images are representative of the expression pattern observed in cells from three independent experiments. The merge images represent the superposition of EGFP-Rev and DAPI, and the white bars represent a length of 10 μM. (**C**) The calculated Fn/c ratios were expressed as means ± SD from three independent experiments, in which 10 cells were analyzed for each of them (*n* = 30). (**D**) The nuclear export activities of EGFP-CAEV Rev WT and mutants M1 to M6 were determined using a CAT reporter assay. HeLa cells were cotransfected with pDM148 and pEGFP-C1 or pEGFP-CAEV Rev WT or pEGFP-CAEV Rev mutants or were untransfected (Unt). Following 48 h of transfection, 50 μg of total cell lysate was used for the assay and the CAT expression levels were normalized via immunoblotting using EGFP-specific antibody (bottom of the panel). The Rev activity was expressed as the ratio of EGFP-CAEV Rev WT or mutant protein CAT expression to the basal expression of EGFP alone. The results represent the mean values ± SD of three separate experiments (triplicate samples per experiment). Antibody against GADPH was used as a loading control. According to one-way ANOVA followed by Dunnett’s test, the values significantly different from those of the CAEV Rev WT protein are indicated by *** (*p* < 0.0005).

In order to identify the aa region necessary for nuclear localization of the CAEV Rev protein, six CAEV Rev deletion mutant proteins fused to EGFP (M1 to M6) were generated (Figure 2A). BoMac cells were used in these experiments because they are of the monocyte/macrophage cell lineage and thus are similar to the target cells for CAEV infection in vivo [1,16]. The cells were transfected with constructs encoding each of the mutant Rev proteins in the absence of LMB. The subcellular distribution of the mutant Rev proteins, which were expressed at comparable levels as determined by Western blot (bottom section of Figure 2D), was analyzed by confocal laser scanning microscopy. Mutants M1, M2 and M6 showed cytoplasmic/nuclear localization and strong nucleolar accumulation (Figure 2B) associated with Fn/c ratios (Figure 2C), similar to that of the CAEV Rev WT protein. In contrast, mutant M3 (deletion of aa 56 to 75) was only observed in the cytoplasm, suggesting the presence of a NLS in the sequence deleted in this mutant protein. Finally, the nuclear accumulation observed with mutants M4 and M5 is consistent with the presence of a NES between aa 76 and 115.

### 3.4. Nuclear Export Activity of EGFP-CAEV Rev Deletion Mutant Proteins

Following analyses of the subcellular localization of CAEV Rev WT and each of the Rev deletion mutants, the effect of mutations on the protein nuclear export activity proteins was examined by using a nuclear export assay. This assay, conducted in Hela cells, relies on the use of a plasmid construct containing the CAEV RRE present within an intron flanked by HIV-1 splice sites and a CAT reporter gene under the control of Simian virus 40 promotor and enhancer [23]. Upon cell transfection with this construct, the nuclear export activity of the CAEV Rev protein can be assessed by expression of the CAT protein. Only traces of CAT (background activity) can be detected in the absence of a functional Rev protein. As shown in Figure 2D, mutants M1, M2 and M6 had nuclear export activity comparable to that of the CAEV Rev WT protein. Mutant M3, which displayed no nuclear localization, and mutants M5 and M6, which only showed nuclear localization, all had a significant decrease in their nuclear export activity when compared to that of the CAEV Rev WT protein. Indeed, the nuclear export activity data obtained for mutants M3, M5 and M6 were similar to those of the negative observed in the untransfected cells and in cells transfected with a plasmid expressing EGFP alone, which were used as negative controls.

### 3.5. The 57 to 82 aa Sequence of the CAEV Rev Protein is Sufficient to Translocate Heterologous Proteins to the Nucleus

**Figure 3:**
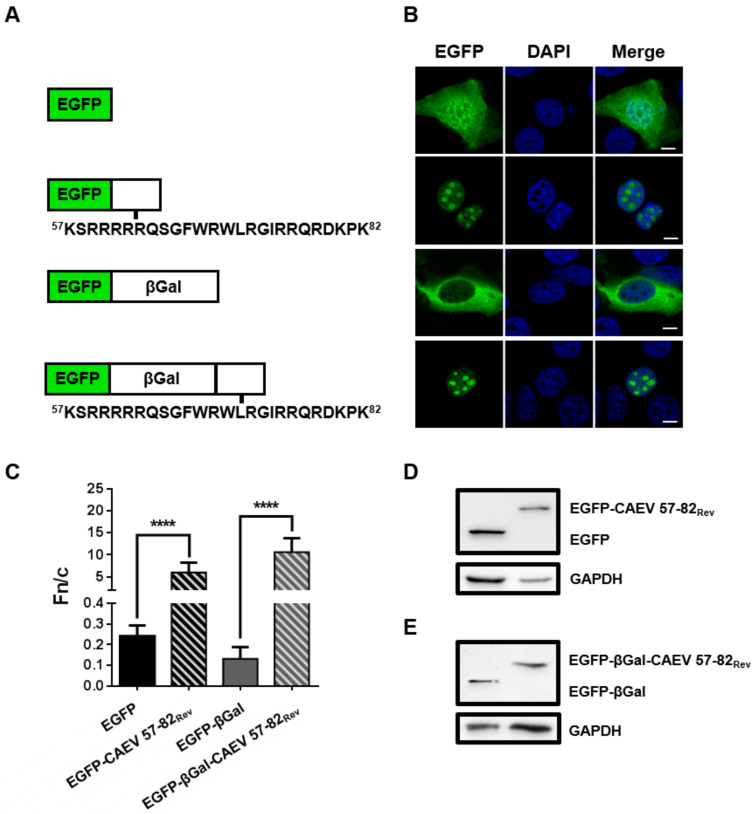
The region between amino acids 57 to 82 of the CAEV Rev protein promotes nuclear localization. (**A**) The sequence aa 57 to 82 of the CAEV Rev protein (CAEV 57–82_Rev_) was cloned into pEGFP-C1 or pEGFP-βGal. (**B**) BoMac cells were transfected with the plasmid constructs encoding for EGFP or EGFP-βGal alone or fused to CAEV 57-82_Rev_. Following 24 h of incubation, cells were fixed and nucleus was counterstained with DAPI, as seen in blue. Expression of EGFP, as seen in green, was visualized by CLSM at 60× magnification. The images are representative of the expression pattern observed in cells from three independent experiments. The merge images represent the superposition of EGFP and DAPI, and the white bars represent a length of 10 μM. (**C**) The calculated Fn/c ratios were expressed as means ± SD from three independent experiments, in which 10 cells were analyzed for each of them (*n* = 30). Significant differences, determined using Student’s T-test, between EGFP or EGFP-βGal alone or fused to the CAEV 57–82_Rev_ sequence, are indicated by **** (*p* < 0.0001). Expression of EGFP (**D**) or EGFP-βGal (**E**) alone or fused to CAEV 57–82_Rev_ were visualized via immunoblotting using EGFP-specific antibody. Antibody against GADPH was used as a loading control.

To show by another means that the region deleted (aa 56 to 75) in mutant M3 contains aa associated to a NLS function, the aa 57 to 82 sequence of the CAEV Rev WT was amplified and cloned in plasmids to generate the EGFP (EGFP-CAEV 57–82_Rev_) and EGFP-βGal (EGFP-βGal-CAEV 57–82_Rev_) proteins fused each to that putative NLS-containing sequence. It is noteworthy that aa 56 (an asparagine residue) deleted in mutant M3 was omitted in this experiment, whereas aa 76 to 82, which are mainly basic residues and thus can be considered as part of a NLS, were included in the sequence to be tested. As shown in Figure 3B,C, EGFP alone showed diffuse distribution in the cytoplasm and the nucleus of transfected BoMac cells, with no nucleolar localization. The addition of the CAEV Rev aa 57 to 82 sequence was sufficient to promote the nuclear/nucleolar accumulation of the EGFP protein. To ensure that the localization of EGFP was not due to passive diffusion of the protein but rather was the result of an active transport, a protein of a larger size, namely EGFP-βGal, was used (~143 kDa as a monomer and ~560 kDa in the tetrameric active form of βGal). As expected, the EGFP-βGal protein alone showed an exclusive cytoplasmic localization. In contrast, the EGFP-βGal-CAEV 57–82_Rev_ accumulated exclusively in the nucleus and the nucleoli of transfected cells (Figure 3B,C). Combined, the results indicate that the aa 57 to 82 sequence of the CAEV Rev protein promoted nuclear localization of heterologous cytoplasmic proteins.

### 3.6. CAEV Rev NLS Mapped between aa 57 and 75

**Figure 4:**
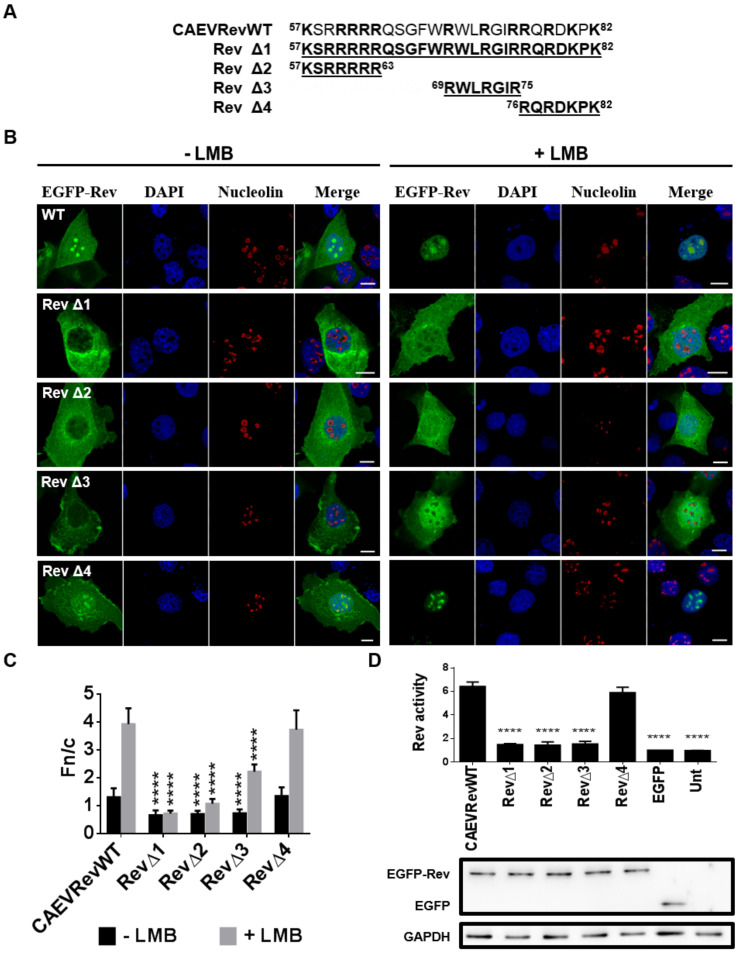
The region encompassing amino acids 57 to 75 is associated with nuclear localization of the CAEV Rev protein. (**A**) CAEV Rev deletion mutants (RevΔ1 to RevΔ4) were generated from pEGFP-CAEV Rev WT using Gibson assembly mastermix. (**B**) Microscopic analyses of the CAEV Rev mutants RevΔ1 to RevΔ4 compared to the CAEV Rev WT. BoMac cells were transfected with pEGFP-CAEV Rev WT or each of the pEGFP-CAEV Rev mutants for 24 h and were treated, where mentioned, with 5 nM of leptomycin B (LMB) 5 h prior to cell fixation and permeabilization. Cells were then immunostained for nucleolin detection, as seen in red, and nucleus was counterstained with DAPI, as seen in blue. Expression of EGFP-Rev, as seen in green, was visualized by CLSM at 60× magnification. The images are representative of the expression pattern observed in cells from three independent experiments. The merge images represent the superposition of EGFP-Rev and DAPI, and the white bars represent a length of 10 μM. (**C**) The calculated Fn/c ratios were expressed as means ± SD from three independent experiments, in which 10 cells were analyzed for each of them (*n* = 30). (**D**) The nuclear export activities of EGFP-CAEV Rev WT and mutants RevΔ1 to RevΔ4 were determined using a CAT reporter assay. HeLa cells were cotransfected with pDM148 and pEGFP-C1 or pEGFP-CAEV Rev WT or pEGFP-CAEV Rev mutants or were untransfected (Unt). Following 48 h of transfection, 50 μg of total cell lysate was used for the assay and the CAT expression levels were normalized via immunoblotting using EGFP-specific antibody (bottom of the panel). The Rev activity was expressed as the ratio of EGFP-CAEV Rev WT or mutant protein CAT expression to the basal expression of EGFP alone. The results represent the mean values ± SD of three separate experiments (triplicate samples per experiment). Antibody against GADPH was used as a loading control. According to one-way ANOVA followed by Dunnett’s test, the values significantly different from those of the CAEV Rev WT protein are indicated by **** (*p* < 0.0001).

In order to identify the region containing residues important for the nuclear localization of the CAEV Rev protein, a second series of deletion mutants was generated (RevΔ1 to RevΔ4) (Figure 4A). As expected, the full NLS deletion mutant protein (RevΔ1; aa 57 to 82) was unable to accumulate in the nucleus/nucleoli, even in the presence of LMB, as shown by the cytoplasmic localization of the protein (Figure 4B,C). The Rev export activity was also lower than that of the CAEV Rev WT protein (Figure 4D). Mutants RevΔ2 and RevΔ3, which contained deletions rich in basic residues (^57^KSRRRRR^63^ and ^69^RWLRGIR^75^, respectively), mislocalized in cells when compared to the CAEV Rev WT protein. Indeed, a faint nuclear accumulation of the proteins was observed in the presence of LMB. Moreover, no nucleolar retention was observed for either mutant protein, regardless of the LMB treatment. In addition, their lower Rev export activity, which was similar to that obtained with the RevΔ1 mutant protein, was consistent with their aberrant localization within the cell (Figure 4D). In contrast, mutant RevΔ4 with a ^76^RQRDKPK^82^ deletion revealed cytoplasmic, nuclear and nucleolar localization and Rev export activity similar to those observed for the CAEV Rev WT protein (Figure 4C,D). Combined, the results showed that the region encompassing aa 57 to 75 of the CAEV Rev protein sequence is associated with NLS function and, as such, correlates with the heterologous protein translocation data obtained above (see Section 3.5).

### 3.7. Identification of the Residues Composing the NLS and NoLS of the CAEV Rev Protein

**Figure 5:**
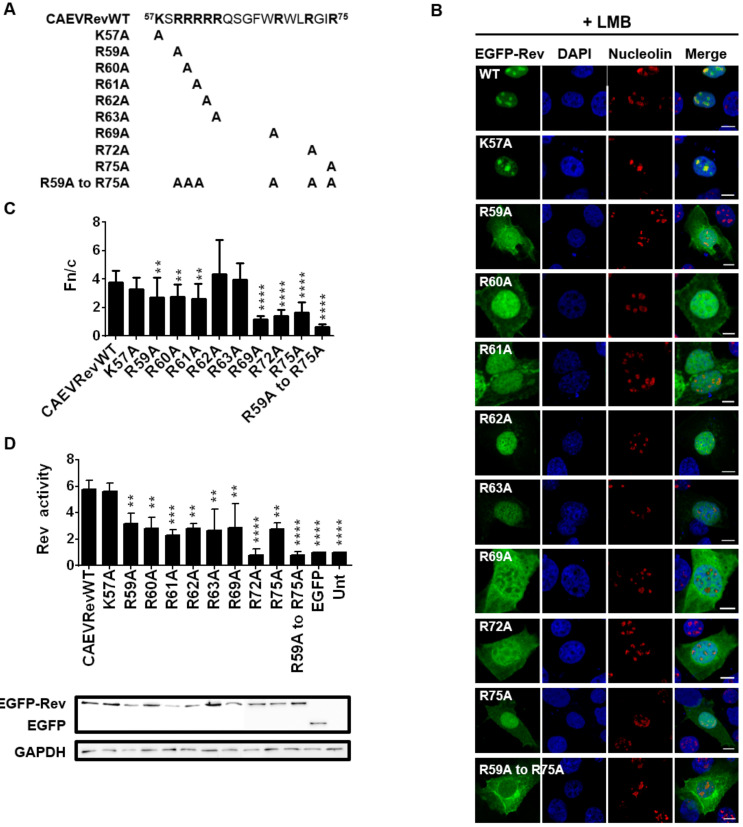
The NLS of the CAEV Rev protein has a monopartite-like structure. (**A**) CAEV Rev lysine (K) or arginine (R) to alanine (A) substitution mutant proteins were generated from pEGFP-CAEV Rev WT by site-directed mutagenesis. (**B**) Microscopic analyses of the CAEV Rev mutant proteins compared to the CAEV Rev WT. BoMac cells were transfected with pEGFP-CAEV Rev WT or pEGFP-CAEV Rev mutants for 24 h and were treated with 5 nM of leptomycin B (LMB) 5 h prior to cell fixation and permeabilization. Cells were then immunostained for nucleolin detection, as seen in red, and nucleus was counterstained with DAPI, as seen in blue. Expression of EGFP-Rev, as seen in green, was visualized by CLSM at 60× magnification. The images are representative of the expression pattern observed in cells from three independent experiments. The merge images represent the superposition of EGFP-Rev, nucleolin and DAPI, and the white bars represent a length of 10 μM. (**C**) The calculated Fn/c ratios were expressed as means ± SD from three independent experiments, in which 10 cells were analyzed for each of them (*n* = 30). (**D**) The nuclear export activities of EGFP-CAEV Rev WT and mutant proteins were determined using a CAT reporter assay. HeLa cells were cotransfected with pDM148 and pEGFP-C1 or pEGFP-CAEV Rev WT or pEGFP-CAEV Rev mutants or were untransfected (Unt). Following 48 h of transfection, 50 μg of total cell lysate was used for the assay and the CAT expression levels were normalized via immunoblotting using EGFP-specific antibody (bottom of the panel). The Rev activity was expressed as the ratio of EGFP-CAEV Rev WT or mutant protein CAT expression to the basal expression of EGFP alone. The results represent the mean values ± SD of three separate experiments (triplicate samples per experiment). Antibody against GADPH was used as a loading control. According to one-way ANOVA followed by Dunnett’s test, the values significantly different from those of the CAEV Rev WT protein are indicated by ** (*p* < 0.005), *** (*p* < 0.0005) and **** (*p* < 0.0001).

**Figure 6:**
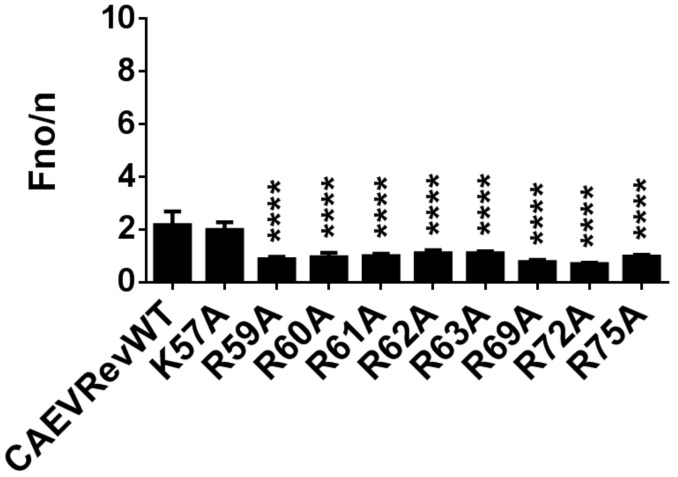
Impact of single alanine substitutions on the nucleolar localization of the CAEV Rev protein. Single alanine substitutions targeting arginine (R) and lysine (K) were generated by site-directed mutagenesis. BoMac cells were transfected with non mutated or mutated pEGFP-CAEV Rev constructs and were treated with 5 nM of leptomycin B (LMB) 5 h prior to cell fixation and immunostaining. Cells were then analyzed by CLSM, as shown in Fig. 5B. The calculated Fno/n ratios were expressed as means ± SD from three independent experiments, in which 10 cells were analyzed for each of them (*n* = 30). Significant differences, using a one-way ANOVA followed by a Dunnett’s test, are indicated by: **** (*p* < 0.0001).

To determine which aa are necessary for the NLS function of the CAEV Rev protein, a series of single alanine substitution mutants targeting basic residues were generated (Figure 5A). BoMac cells were then transfected with the various plasmid constructs and the localization of the mutant proteins was compared to that of the CAEV Rev WT protein in the presence of LMB. The alanine substitution at residue 57 (K57A) had no impact on the mutant subcellular localization, which was similar to that of the CAEV Rev WT protein. Indeed, LMB treatment resulted in complete nuclear/nucleolar accumulation of the mutated protein, suggesting the presence of functional NLS and NoLS in mutant K57A (Figure 5B,C and Figure 6). The Rev export activity for this mutant was also similar to that of the CAEV Rev WT protein (Figure 5D). In contrast, each of the R to A substitutions at residues 59, 60, 61, 69, 72 and 75 resulted in a decrease in the nuclear accumulation of the mutant proteins which were observed in the cytoplasm, even in presence of LMB (Figure 5B). These observations were supported by significantly lower calculated Fn/c ratios (Figure 5C). In accordance with this result, the Rev export activity of these mutant proteins was significantly lower than that of the CAEV Rev WT protein (Figure 5D). It is noteworthy that none of the mutant proteins did accumulate in the nucleoli (Figure 5B), an observation that is supported by their lower Fno/n ratios (Figure 6) when compared to the CAEV Rev WT protein. This suggests that the 59, 60, 61, 69, 72 and 75 arginines mutated in these proteins also are at play in the NoLS function of the CAEV Rev protein in addition to that of the NLS. These results were confirmed by the generation of a multiple alanine substitution mutant targeting these aa (mutant R59A to R75A). As visualized in Figure 5, both the nuclear/nucleolar localization and the functional activity of the CAEV Rev mutated protein were completely abolished. Regarding mutants R62A and R63A, they showed subcellular distribution similar to that of the CAEV Rev WT with Fn/c ratios similar to that of the CAEV Rev WT protein. However, these mutants were not able to accumulate in the nucleoli, as determined by the microscopy images (Figure 5B) and the Fno/n ratios (Figure 6). Impaired Rev export activity was also noted, which is additional evidence of the mislocalization of the mutant proteins. These results showed that the 62 and 63 arginine residues are not associated with nuclear but rather with nucleolar localization. Altogether, the results indicated that the NLS of the CAEV Rev protein is composed of residues ^59^RRR-RWLRGIR^75^ with a monopartite-like structure. With the generated results, we also identified the presence of a NoLS that was found to be partly associated with the NLS. Indeed, the NoLS is composed of arginine residues within the ^59^RRRRR-RWLRGIR^75^ sequence. Therefore, the NoLS-associated residues correspond to all aa composing the NLS in addition to the arginines at residue positions 62 and 63 in the protein.

### 3.8. The CAEV Rev NES

**Figure 7:**
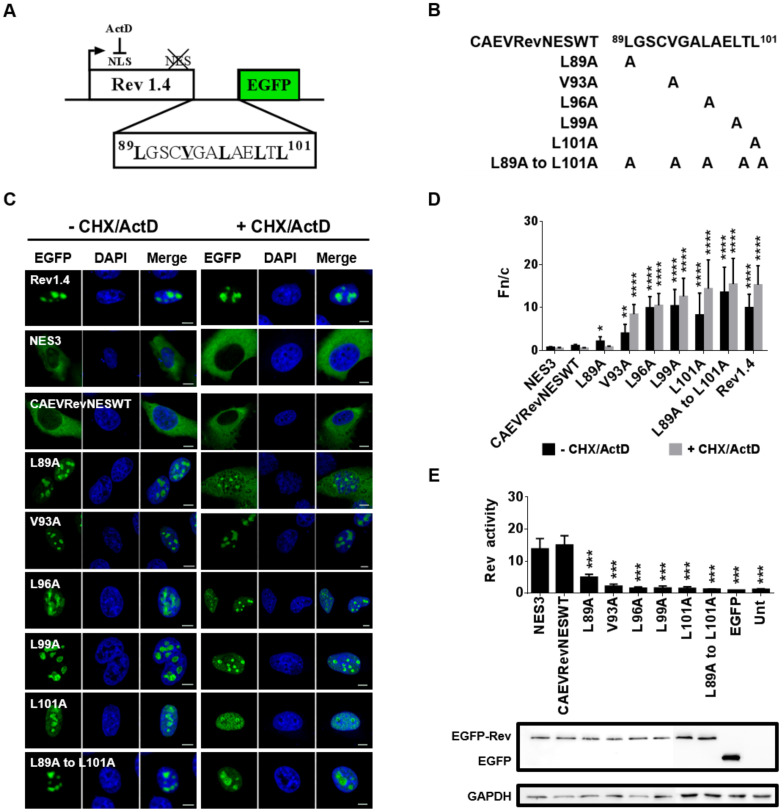
The NES of the CAEV Rev protein lies between amino acids 89 to 101. (**A**) Plasmids encoding HIV-1 Rev(1.4)-EGFP (negative control; Rev1.4), HIV-1 Rev(1.4)-NES3-EGFP (positive control; Rev1.4NES3) or plasmids encoding HIV-1 Rev(1.4) containing either the predicted NES sequence (amino acids 89 to 101) of the CAEV Rev WT protein (CAEVRevNESWT) or (**B**) each of the CAEV NES mutated sequences were used. (**C**) HeLa cells were transfected for 24 h and were incubated with or without CHX (10 μg/mL) and ActD (5 μg/mL) 3 h prior to cell fixation. Cells were then fixed and counterstained with DAPI, as seen in blue. Expression of EGFP-Rev, as seen in green, was visualized by CLSM at 60× magnification. The images are representative of the expression pattern observed in cells from three independent experiments. The merge images represent the superposition of EGFP-Rev and DAPI, and the white bars represent a length of 10 μM. (**D**) The calculated Fn/c ratios were expressed as means ± SD from three independent experiments, in which 10 cells were analyzed for each of them (*n* = 30). (**E**) The nuclear export activity of the EGFP-fused HIV-1 Rev protein was determined using a CAT reporter assay. HeLa cells were cotransfected with pDM128 and pRev(1.4)-EGFP or pRev(1.4)-NES3-EGFP or plasmids containing the NES sequence of CAEV Rev WT or each of the CAEV Rev NES mutated sequences or were left untransfected (Unt). Following 48 h of transfection, 50 μg of total cell lysate was used for the assay and the CAT expression levels were normalized via immunoblotting using EGFP-specific antibody (bottom of the panel). Rev activity was then determined as the ratio of HIV-1 Rev protein CAT expression harboring the HIV-1 Rev NES WT (NES3), the CAEV Rev NES WT or the CAEV Rev NES mutant to the basal expression from pDM128 construct co-transfected with empty pEGFP-C1 alone. The results represent the mean values ± SD of three separate experiments (triplicate samples per experiment). Antibody against GADPH was used as a loading control. According to one-way ANOVA followed by Dunnett’s test, the values significantly different from those of CAEV Rev WT protein are indicated by * (*p* < 0.05), ** (*p* < 0.005), *** (*p* < 0.0005) and **** (*p* < 0.0001).

The CRM1 protein exports proteins to the cytoplasm by interacting with a short region within the protein termed the NES. Despite the fact that NESs usually are leucine-rich regions, they can also be composed of other hydrophobic amino acids (e.g., valine, methionine and phenylalanine) [26,32,33]. Since this study revealed that the CAEV Rev protein uses the CRM1 pathway for its nuclear export (Figure 1), it was therefore relevant to identify the aa composing its NES. As mentioned above, the deletion of aa 76 to 115 in mutants M4 and M5 greatly disrupted the nuclear export of the Rev protein, suggesting the presence of a NES in this region. This also correlated with a predicted NES CRM1-dependent signal from aa 89 to 101 of the CAEV Rev protein, as determined by using the NetNES 1.1 prediction program [34]. A putative NES can be analyzed using the HIV-1 Rev(1.4)-EGFP nuclear export assay by which the nuclear export of the HIV-1 Rev protein is evaluated [26]. This assay is based on the use of the HIV-1 pRev(1.4)-EGFP plasmid, which encodes the HIV-1 Rev protein fused to the EGFP protein and devoid of its functional NES. Therefore, the HIV-1 EGFP fusion protein will accumulate in the nucleus/nucleoli of cells transfected with the plasmid (see Rev1.4 in Figure 7C). By inserting a functional NES between the HIV-1 Rev(1.4) and the EGFP, as illustrated in Figure 7A, the nucleocytoplasmic shuttling activity of the fusion protein can be restored.

The nucleic acid sequence coding for the predicted NES (aa 89 to 101) of the CAEV Rev protein was inserted into the pRev(1.4)-EGFP plasmid (Figure 7A). In addition, the hydrophobic aa within the putative NES were targeted for single alanine substitution mutations (Figure 7B). HeLa cells were then transfected with each of the constructs (CAEVRevNESWT or the mutated CAEV NESs) and were treated with CHX and ActD 3 h prior to cell fixation or were left untreated, as described elsewhere [28]. CHX inhibits protein synthesis and was used to ensure that any cytoplasmic green fluorescence resulted from the nuclear export of EGFP fusion protein rather than novel synthesis of the protein. ActD was used to promote the re-localization of HIV-1 Rev in the cytoplasm [9]. The Fn/c ratios were used to evaluate the strength of each NES under study (CAEVRevNESWT and each of the mutated CAEV NES). As mentioned above, the HIV-1 Rev(1.4)-EGFP protein (Rev1.4), which was used as a negative control, localized mainly in the nucleus and the nucleoli of transfected cells. In contrast, the HIV-1 Rev(1.4)-NES3-EGFP protein (NES3), which contains the whole HIV-1 Rev NES, was mainly cytoplasmic (Figure 7C). By inserting the CAEV Rev WT predicted NES sequence into the pRev(1.4)-EGFP vector (CAEVRevNESWT), the transfected cells treated with or without CHX/ActD showed cytoplasmic localization of the fusion protein associated with low calculated Fn/c ratios (Figure 7C,D). Alanine substitution of each of the hydrophobic residues within the CAEV Rev WT predicted NES greatly disrupted the nuclear export of the mutant proteins listed in Figure 7B (mutants L89A, V93A, L96A, L99A and L101A) as their subcellular localization, regardless of the CHX/ActD treatment, was mainly nuclear and nucleolar (Figure 7C). It is noteworthy that mutant L89A showed different subcellular distribution from the other mutant proteins in CHX/ActD-treated cells with cytoplasmic and nuclear/nucleolar localization (Figure 7B,C). The observed nuclear/nucleolar subcellular localization suggests that the L89A mutation did impair, to a certain degree, the CAEV Rev NES function. These results were confirmed by a nuclear export assay using the pRRE-HIV plasmid containing the CAT reporter gene (Figure 7E) and where the HIV-1 Rev export activity was significantly lower for all single alanine substitution mutants when compared to that of the CAEV Rev NES (CAEVRevNESWT) and the HIV-1 Rev NES (NES3). Multiple alanine substitutions targeting all hydrophobic residues (mutant L89A to L101A) showed a higher Fn/c ratio and lower Rev activity when compared to the other proteins, in addition to a nuclear/nucleolar accumulation of the protein. Combined, the results indicate that residues ^89^L, ^93^V, ^96^L, ^99^L and ^101^L are essential for the CAEV Rev NES function.

## 4. Discussion

To ensure their function, certain proteins must shuttle between the cytoplasm and the nucleus through nuclear pore complexes (NPCs). Smaller molecules (~40 kDa) usually travel across the nuclear membrane by passive diffusion, whereas larger proteins shuttle in an active means [35,36,37]. Proteins that are imported or exported through NPCs in an energy-dependent manner contain at least one NLS and a NES [38,39]. The lentiviral Rev as well as retroviral Rev-like proteins (such as Rem (mouse mammary tumor virus), Rec (human endogenous retrovirus K) and Rex (human T-cell leukemia virus type 1) proteins) are no exception [8,27,40,41,42,43,44]. These regulatory proteins mediate the nucleocytoplasmic transport of viral RNAs and, to exert their function, travel across NPCs through the interaction of their NLS and NES with host cellular proteins [45].

It was previously shown that the CAEV Rev protein localizes in the cytoplasm and the nucleus of virus-infected cells, with accumulation into the nucleoli [16]. Similar subcellular localization was described for the Rev protein of the Visna virus, a small ruminant lentivirus like CAEV [46]. In this study, the subcellular distribution was assessed in cells transiently transfected with a plasmid construct encoding the CAEV Rev protein in fusion with EGFP. It was shown that the subcellular distribution of the CAEV Rev-EGFP fusion protein was similar to that seen previously for the native Rev protein in CAEV-infected cells with cytoplasmic and nuclear localization of the former protein with accumulation in the nucleoli (Figure 1). Such nucleolar accumulation has been reported for the Rev proteins of other lentiviruses, such as the human immunodeficiency virus type 1 (HIV-1), bovine immunodeficiency virus (BIV), Jembrana disease virus (JDV) and feline immunodeficiency virus (FIV), in cells either transfected with an adequate plasmid construct and/or infected with the virus [28,47,48,49].

The aa sequence region necessary for the localization of the CAEV Rev protein in the nucleus as well as the key aa involved in the nuclear export of the protein were identified. By using several deletion mutants, it was shown that deletion of aa 56 to 75 (mutant M3) greatly impaired the nuclear localization, with predominantly cytoplasmic localization of the protein. It was also observed that the nuclear export of the CAEV Rev protein was disrupted by the deletion of aa 76 to 95 and aa 96 to 115 (mutants M4 and M5, respectively), with nucleus/nucleolus localization of the mutant proteins. In addition, the export activity of mutants M3, M4 and M5 was significantly lower than that of the CAEV Rev WT protein (Figure 2). To investigate further and to confirm that the region deleted in mutant M3 was associated with NLS function, the region that encompasses aa 57 to 82 was fused to the C-terminus of EGFP and EGFP-βGal. The aa 76 to 82 from the CAEV Rev WT protein were added in the construction to include basic residues in proximity to the region deleted in M3 (aa 56 to 75) since NLSs are known to be rich in arginines, lysines or other basic residues [11]. The results showed that this region promoted the relocalization of cytoplasmic proteins to the nucleus and nucleoli of transfected cells (Figure 3). In order to identify the important aa involved in NLS function, additional deletion as well as single arginine to alanine substitution mutants were generated in the CAEV Rev protein targeting aa 57 to 82. The results showed that the CAEV Rev protein has a monopartite-like NLS composed of ^59^RRR-RWLRGIR^75^. Thus, the NLS of the CAEV Rev protein is exclusively composed of arginine residues which are grouped into two clusters separated by seven aa. To be categorized as bipartite NLS, the two clusters must be separated by a minimum of eight aa [12]. Thus, the NLS sequence of the CAEV Rev protein would belong to a monopartite-like type. In order to validate its monopartite nature, additional studies involving importin α are necessary since monopartite-type NLS binds directly with this karyopherin to trigger the classical import pathway [11]. Regardless, the NLS of the CAEV Rev protein resembles those of the HIV-1 [50] and JDV [28] Rev proteins, which also are exclusively composed of arginine residues and have a monopartite-like structure. It is noteworthy that the single arginine to alanine substitution at positions 62 and 63 did not affect the nuclear localization of the CAEV Rev protein. However, these mutations impaired the nucleolar localization and the functional activity of the mutant proteins. Moreover, each mutated aa composing the NLS resulted in no nucleolar localization of the mutant proteins. According to these results, we concluded that the region composed of ^59^RRRRR-RWLRGIR^75^ in the CAEV Rev protein is associated with NoLS function and is intrinsically associated for its major part with the sequence that composes the NLS of the protein.

All lentiviral Rev proteins are dependent on the interaction with the karyopherin CRM1 for their nuclear export [25,28,30,48]. The CAEV Rev protein’s nuclear export is no exception. As shown in this study, the nuclear export of the protein was completely abolished in LMB-treated cells (Figure 1). LMB inhibits the interaction between CRM1 and the hydrophobic aa-rich NES, thus blocking the export to the cytoplasm of proteins harboring a CRM1-dependent NES [31]. This LMB result prompted us to identify the aa composing the NES of the CAEV Rev protein. As mentioned before, mutants M4 and M5 (in which aa 76 to 115 have been deleted) revealed cellular mislocalization when compared to the CAEV Rev WT protein. The nuclear/nucleolar accumulation of these mutants suggested the presence of a NES in the region deleted in these mutants. It was predicted that aa 89 to 101, a sequence rich in leucines, could serve as a NES. This sequence was then analyzed with the Rev(1.4)-EGFP nuclear export assay. When inserted into the HIV-1 Rev(1.4)-EGFP plasmid, the ^89^LGSCVGALAELTL^101^ sequence was able to restore the nuclear export activity of the HIV-1 Rev protein (Figure 7). In order to identify the key residues associated with the NES, single or multiple alanine substitution mutations targeting the hydrophobic residues within the aa 89 to 91 sequence were generated. The results obtained from the Rev(1.4)-EGFP and Rev activity CAT assays revealed that ^89^L, ^93^V, ^96^L, ^99^L and ^101^L were important in the NES function. Thus, it could be concluded that the NES of the CAEV Rev protein is composed of ^89^**L**GSC**V**GA**L**AE**L**T**L**^101^, where key residues of the NES are indicated in bold.

Several cellular and viral proteins contain a NES that interacts directly with CRM1 [51]. CRM1 recognizes four key hydrophobic residues in the NES (noted Φ^1^–Φ^4^) that will bind to its hydrophobic pockets. NESs are arranged in many different ways and are now described with the Φ^1^-(X)_2–3_-Φ^2^-(X)_2–3_-Φ^3^-(X)-Φ^4^ consensus sequence, where Φ refers to L, V, F, M or I and X to any other aa [52]. In a more recent study, it was shown that optimal NES binding to CRM1 can be achieved by the interaction of a fifth residue, noted as Φ^0^ [32]. The original NES consensus sequence classification was based on the first proteins identified as carrying a NES that interacts specifically with CRM1: the cyclic-AMP-dependent protein kinase inhibitor (PKI) protein and the HIV-1 Rev protein [26]. As seen in Table 1, proteins harboring Φ^0^XXΦ^1^XXXΦ^2^XXΦ^3^XΦ^4^ and Φ^0^Φ^1^XΦ^2^XXΦ^3^XΦ^4^ consensus sequences belong to the PKI NES class and HIV-1 NES class, respectively. The HIV-1 Rev protein is the only representative of its class among the NESs of Rev/Rev-like proteins. In contrast, other lentiviral Rev protein NESs have been identified as being of the PKI NES class, like those of the BIV and JDV Rev proteins [9,28]. As mentioned above, the ^89^L, ^93^V, ^96^L, ^99^L and ^101^L residues are critical for nuclear export of the CAEV Rev protein. Thus, the CAEV Rev NES presents a Φ^0^XXXΦ^1^XXΦ^2^XXΦ^3^XΦ^4^ consensus sequence (Table 1) with the presence of a third aa between the Φ^0^ and Φ^1^ hydrophobic residues, which is unique among lentiviral Rev proteins.

It has been previously shown that the Visna virus encodes for a post-transcriptional activator of viral gene expression, namely the Visna Rev protein. Like CAEV Rev, Visna Rev is essential for viral replication due to its function in the export of partially or unspliced RNAs in the cytoplasm. The protein also harbors a domain rich in basic aa and another rich in hydrophobic aa, which act like a NLS and a NES, respectively [15,53]. Since the sequences of SRLVs are highly conserved, as shown in Supplementary File S1, one may suggest that the NLS/NoLS- and NES-associated activities of the Visna Rev protein are likely analogous to the CAEV Rev protein.

It is well recognized that several viruses induce important alterations of the nucleoli which may impact the outcome of infection, such as viral replication, virus assembly and the control of intracellular trafficking [54,55]. We previously reported that the BIV Rev protein interacts with nucleolar phosphoprotein B23 in cells and this interaction has a positive impact on the virus replication. It was also shown that the NLS/NoLS were essential and sufficient to mediate the BIV Rev–B23 interaction [56]. Whether such NLS/NoLS-dependent Rev protein–B23 interaction would occur in CAEV has yet to be investigated.

In conclusion, this study revealed that the nucleocytoplasmic transport of the CAEV Rev protein is mediated by NLS and NES motifs. Taken together, these findings will deepen the knowledge about the mechanisms and modes of action of viral proteins during lentiviral infection and may be taken into account for future therapy development to combat lentiviruses.

## Figures and Tables

**Table 1:** Optimal NES consensus sequences of different Rev proteins.

Protein	NES ^1^	References
PKI-type NESs		
Consensus	**Φ^0^**XX**Φ^1^** XXX**Φ^2^** XX**Φ^3^**X**Φ^4^**	
PKI	**I**NE**L**ALK**L**AG**L**D**I**	[32]
JDV Rev	**M**AE**L**EER**F**ED**L**A**L**	[28]
BIV Rev	**I**QQ**L**EDL**V**RH**M**S**L**	[9]
HIV-1 Rev-type NES		
Consensus	**^0^Φ^1^** X**Φ^2^** XX**Φ^3^**X**Φ^4^**	
HIV-1 Rev	LQ**LP**P**L**ER**L**T**L**	[32]
CAEV Rev NES		
Consensus	**Φ^0^**XXX**Φ^1^** XX**Φ^2^** XX**Φ^3^**X**Φ^4^**	This study
CAEV Rev	**L**GSC**V**GA**L**AE**L**T**L**	

^1^ The hydrophobic residues composing the NES are shown in bold.

## Supplementary Materials

The following are available online at https://www.mdpi.com/1999-4915/12/8/900/s1, Supplementary File S1: Multiple amino acid sequence alignment of Rev proteins from SRLV strains.
