# Supplementary material for: Characterization of Signal Sequences Determining the Nuclear/Nucleolar Import and Nuclear Export of the Caprine Arthritis-Encephalitis Virus Rev Protein
Source: Viruses. 2020 Aug 17;12(8):900. doi: 10.3390/v12080900 (PMC7471974; doi:10.3390/v12080900)
Supplement: Supplementary file 1 [file viruses-12-00900-s001.zip › viruses-883507-supplementary.pdf]

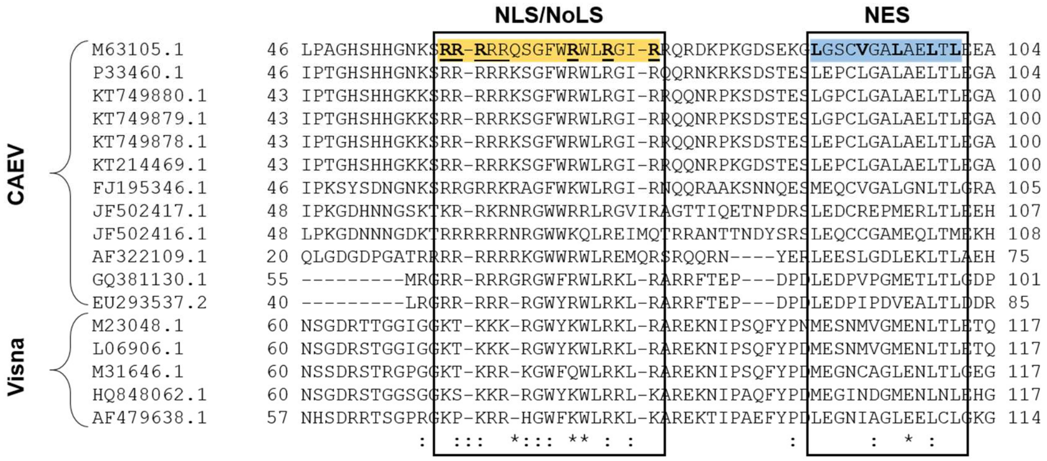

Supplementary File S1. Multiple amino acid sequence alignment of Rev proteins from SRLVs strains. Multiple sequence alignments using MFFAT (Multiple Alignment using Fast Fourier Transform, v7.429) were done using several CAEV and Visna strains. NLS key-residues are indicated in bold while those composing the NoLS are underlined. Hydrophobic residues composing the NES are shown in bold. This study was carried out with M63105.1 CAEV Cork strain as the reference sequence. The letters/numbers in the left column correspond to the accession numbers found in GenBank.
